# First report of tilapia lake virus emergence in fish farms in the department of Córdoba, Colombia

**DOI:** 10.14202/vetworld.2021.865-872

**Published:** 2021-04-10

**Authors:** Héctor Contreras, Adriana Vallejo, Salim Mattar, Luis Ruiz, Camilo Guzmán, Alfonso Calderón

**Affiliations:** 1 Institute of Biological Research of the Tropic, University of Córdoba, Colombia; 2 Aquatic Health and Water Quality laboratory, Aquaculture Program, University of Córdoba, Colombia; 3 Department of Pharmacy, Faculty of Health Sciences, University of Córdoba, Colombia

**Keywords:** alternative, animal use, developing countries, disease outbreaks, economic factor, fish diseases, sentinel surveillance

## Abstract

**Background and Aim:**

In 2016, the tilapia-producing farms in the department of Córdoba, Colombia, had witnessed outbreaks of disease with clinical signs compatible with those caused by the tilapia lake virus (TiLV). This study was conducted to confirm the presence of TiLV in some fish farms in the department of Córdoba.

**Materials and Methods:**

A descriptive cross-sectional study was conducted in seven farms using a non-random sampling method from July 2016 to December 2017. A total of 66 fish, including 33 healthy fish and 33 fish with clinical signs, were caught, from which 178 tissue samples of spleen, liver, and brain were collected. RNA was extracted from each organ using TRIzol^®^. cDNA was synthesized using a retrotranscriptase and a universal amplification primer. The polymerase chain reaction was performed using primers specific to TiLV, in which the primers were amplified in a 491 bp region in segment 3 of TiLV, and the amplicons were sequenced using the Sanger method.

**Results:**

Of the seven farms surveyed, 3 (42.85%) had TiLV in the collected fish. Of the 66 collected fish, 18 (27.27%) were infected with TiLV. The virus was detected in the brain (64.3%, 18/28), spleen (61.9%, 13/21), and liver (35.7%, 10/28). The sequences were recorded in GenBank with the codes MH338228, MH350845, and MH350846. Nucleotide homology analyses revealed that this study’s circulating strains exhibited 97% identity with the Israeli strain (GenBank KU751816.1).

**Conclusion:**

This is the first official report of TiLV in the department of Córdoba, Colombia. The circulating strains detected in this study exhibited 97% identity with the Israeli strain.

## Introduction

Tilapia (*Oreochromis* spp.) is considered the second most important species in aquaculture worldwide. The annual global production is estimated at 6.4 million metric tons valued at more than USD 9.8 billion, and it is expected that by the year 2030, the production will increase to 7.3 million metric tons [1]. China, Ecuador, Egypt, Indonesia, and Thailand were the producers of this species, whose production was valued at USD 1.8 trillion in 2015. The United States has the top imports of this fish and consumes approximately 225 metric tons annually [2].

Tilapia is an economical source of protein, primarily in developing countries [3,4]. It is considered a fast-growing fish, relatively resistant to diseases [5], although its production is threatened by some pathogenic microorganisms [6-8]. Massive deaths due to infectious etiologies have resulted in substantial economic losses [9]. Diseases occurring in fish farmed for commercial purposes have been primarily attributed to crop intensification; increase in chemical, biological, and physical factors; and stress triggering agents that influence fish physiology [10].

In 2014, a highly contagious emerging virus was reported for the 1^st^ time in Israel that was associated with mortality levels of >80% in *Oreochromis* spp. This virus was termed the tilapia lake virus (TiLV) [11]. After its discovery, there had been isolated outbreaks with high mortalities in tilapia in fish farms in Ecuador [11,12], Israel [13], Egypt [14], Thailand [9,15], and more recently in India [16], Malaysia [17], Chinese Taipei [18], the Philippines [19], Mexico [20], Peru [21], and the United States [22], with substantial economic losses. Estimates from Egypt indicated a production loss of 98,000 metric tons, at a value of around USD 100 million, due to the “summer mortality” syndrome in 2015, of which TiLV may have played a part [14,23].

TiLV is an RNA virus enveloped in a single segmented strand and a negative sense [11,13,24]. The RNA has 10 segments that code for 10 proteins; all segments have an open reading frame (ORF). Segment 1 is the longest and has ORFs with weak sequence homology to the PB1 subunit of the influenza virus C [25]. None of the other segments exhibit homology with any other known virus [13,25]. However, the conserved terminal regions of 5´ and 3´ are similar to the genomic organization of the Orthomyxoviridae family members [25].

Animals infected with TiLV show clinical signs such as lethargy, paleness, lack of appetite, skin lacerations, eye lesions, hepatic, cephalic, and splenic lesions [11,13,15]. TiLV in diseased fish tissues was reported in Colombia in the year 2017 [12].

Between 2016 and 2017, there were high mortality rates in fish in tilapia-producing farms in the department of Córdoba, Colombia, Caribbean area. The disease exhibited clinical signs compatible with those caused by TiLV, an epidemiological situation due to which the present study was conducted to confirm the presence of TiLV in some fish farms in the department of Córdoba, Colombia.

## Materials and Methods

### Ethical approval

The University of Cordoba follows strict requirements of the legislation on scientific research in biological diversity. Those involve collecting, capturing, hunting, fishing, manipulating the biological resource, and mobilizing in the national territory. The Institute of Biological Research of the Tropic-IIBT requested the Corporation of the Valleys of Sinú and San Jorge-CVS’s local approval dated May 21, 2016.

### Type of study, period, and geographical area

**Figure-1:**
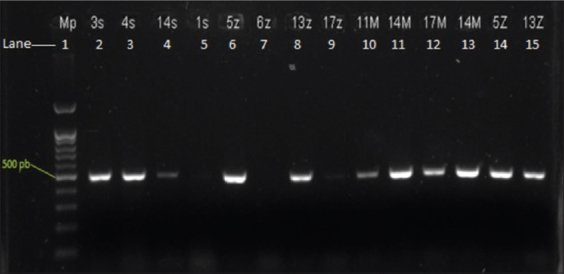
Agarose gels electrophoresis using SYBR safe with TiLV amplicons of Momil 1, Buenavista, and San Bernardo del Viento. Lane 1, molecular weight of 500 bp. Lanes 2-5 show TiLV amplicons from San Bernardo del Viento. Lanes 6, 8, 9, 14, and 15 show TiLV amplicons from Buenavista; lanes 10-13 show TiLV amplicons from Momil. Lane 7, negative sample. TiLV=Tilapia lake virus.

This was a descriptive cross-sectional study conducted using a convenience non-random sampling method from July 2016 to December 2017, a period during which outbreaks of a disease with high mortality of fish occurred in the department of Córdoba, area of the Colombian Caribbean (Figure-1). The study sites were seven fish farms located in Buenavista, Lorica, Sahagún, Momil, San Bernardo del Viento, and Ciénaga de Oro, whose annual average temperature was 28°C, and annual average rainfall was 2000 mm, with August-November being the rainiest months. The same numbers of diseased and healthy tilapia fish were collected from each farm, considering that they were from the same lot and culture pond. The distribution of the specimens was as follows: Momil (n=8), Buenavista (n=10), Lorica (n=10), Sahagún-1 (n=10), Sahagú-2 (n=8), San Bernardo del Viento (n=10), and Ciénaga de Oro (n=10).

### Collection of samples

Between 8 and 10 specimens of tilapia (*Oreochromis* spp.) were collected from each fish farm, resulting in a total of 66 fish comprising 33 healthy fish and 33 fish with clinical signs of disease. The fish were then anesthetized using eugenol (1 ppm) and euthanized by a cervical puncture. The specimens were dissected *in situ*, avoiding cross-contamination between tissues and organs. A total of 178 tissue fragments were distributed as follows: 66 brain samples (Momil [n=8], Buenavista [n=10], Lorica [n=10], Sahagún-1 [n=10], Sahagún-2 [n=8], San Bernardo del Viento [n=10], and Ciénaga de Oro [n=10]); 66 liver samples (Momil [n=8], Buenavista [n=10], Lorica [n=10], Sahagún-1 [n=10], Sahagún-2 [n=8], San Bernardo del Viento [n=10], and Ciénaga de Oro [n=10]); and 46 spleen samples (Momil [n=8], Buenavista [n=10], Lorica [n=5], Sahagún-1 [n=7], Sahagún-2 [n=6], San Bernardo del Viento [n=3], and Ciénaga de Oro [n=7]). Tissues were placed in cryovials containing RNAlater^®^ solution (Invitrogen, USA) and stored at −80°C until processing. To maintain the anonymity of the farms, they were named after the municipality where they were located as Momil, Ciénaga de Oro, Sahagún-1 and -2, San Bernardo del Viento, Buenavista, and Lorica. Similarly, the origin of fingerlings was named after the department of origin (Caldas, Meta, and Córdoba).

### Molecular methods for the detection of TiLV

According to the manufacturer’s instruction, approximately 50 and 100 mg of each tissue samples were taken for RNA extraction using TRIzol^®^ (Invitrogen). The extracted RNA was suspended in nuclease-free ultrapure distilled water and stored at −80°C. The purity and concentration of total RNA were determined in each sample using the NanoDrop^®^ 2000 spectrophotometer (Thermo Scientific, USA). cDNA was synthesized using a retrotranscriptase (M-MLV^®^ Promega, USA) according to the manufacturer’s instruction and a universal primer of amplification (Random^®^ Primer Promega). The conventional polymerase chain reaction was performed using the specific primers nested ext-1 (5´TATGCAGTACTTTCCCTGCC3´) and nested ext-2 (5´TTGCTCTGAGCAAGAGTACC3´) as described by Eyngor *et al*. [13], which amplify a 491 bp fragment of segment 3 of TiLV. Agarose electrophoresis was conducted using SYBR^®^ Safe DNA gel stain (Invitrogen) in 1× TBE buffer. The obtained amplicons were sequenced using the Sanger method. The sequences were edited in the MEGA 6.06 program [26], and the similarity of the nucleotide fragment with the homologous position of the strain genomes available in the NCBI database was determined by BLAST. Consensus alignment of the sequences obtained in this study was performed with the GenBank data. Elaboration of the phylogenetic tree was performed using the Kimura 2-parameter model [26].

## Results

### Prevalence of TiLV in farms

A total of 66 fish were analyzed, including Momil (n=8), Buenavista (n=10), Lorica (n=10), Sahagún-1 (n=10), Sahagún-2 (n=8), San Bernardo del Viento (n=10), and Ciénaga de Oro (n=10), of which 33 fish showed clinical signs of TiLV disease, whereas the remaining 33 fish showed no signs. Among the 66 fish analyzed from seven farms, TiLV was detected in 18 fish (27, 27%) in Momil (8), Buenavista (5), and San Bernardo del Viento (5) (Figure-1), with mortality rates between 30% and 87% (Tables-1 and 2). Of these 18 TiLV-positive fish, 14 had at least one or two clinical signs of disease, and the remaining 4 fish had no apparent clinical signs. In the other two farms (Buenavista and San Bernardo del Viento), only fish with clinical signs of disease were positive for TiLV, which exhibited 50% morbidity (Tables-1 and 2) [13-17,21,24,27-29].

**Table-1:** Dates sampling, geographical descriptions of farms, and PCR results per fish farm analyzed.

Sampling date	Municipality – Farm	Age	GM (%)	Seed’–origin	Population	Sample (N)	AH	CS	Fishes infected PCR (+)	Apparent infection prevalence (%)
July 26, 2016	Momil-1	Ad	85	Caldas	100.000	8	4	4	8/8	100
September 14, 2016	Buenavista	Ad	87	Meta	5.000	10	5	5	5/10	50
November 19, 2016	Lorica	Al	30	Caldas	75.000	10	5	5	0/10	0
April 14, 2017	Sahagún-1	Ad	20	Meta	80.000	10	5	5	0/10	0
April 14, 2017	Sahagún-2	Al	45	Meta	100.000	8	4	4	0/8	0
April 16, 2017	S Bernar del Viento	Al	30	Meta	14.000	10	5	5	5/10	50
June 26, 2017	Cienaga de Oro	Ad	30	Cordoba	100.000	10	5	5	0/10	0
Total	7				1.074.000	66	28	28	18/66	42.85

GM=Gross mortality, AH=Apparently healthy, CS=With clinical signs, Ad=Adult, Al=Fingerlings, PCR=Polymerase chain reaction

**Table-2:** Prevalence and mortality of TiLV in different countries.

Country Year	Positive sampling locations/total	Positive fishes/total sampled	Involved specie	% mortality	References
Israel 2014	25/25	-	*S. galilaeus, T. zilli, O. aureus, T. simonis intermedia* y tilapia híbrida *O. niloticus×O. aureus*	>80	[13]
Egypt 2015	4/8	52/207	*O. niloticus*		[27]
Ecuador 2017	1/1	17/27	*O. niloticus*	90	[24]
Thailand 2017	22/32	ND/325	*(O. niloticus)* and red hybrid tilapia (*Oreochromis* spp.)	20-90	[15]
Egypt 2017	3/11	ND/33	*O. niloticus*	9.2	[14]
India 2018	3/3	ND/45	*O. niloticus*	80-90	[16]
Malaysia 2018	1/1	NR/20	Red hybrid tilapia *(O. niloticus×O. mossambicus)*	25	[17]
Malaysia 2018	1/1	5/5	*O. niloticus, P. schwanenfeldii*	ND	[29]
Uganda 2018	6/14	10/83	*O. niloticus*	ND	[28]
Tanzania 2018	4/4	18/108	*O. niloticus*	ND	[28]
Peru 2019	3/4	4/27	*O. niloticus*	ND	[21]
Colombia 2016-2017	3/7	18/66	Red hybrid tilapia (*Oreochromis* spp.)	30-87	Present study

ND=No data, TiLV=Tilapia lake virus, *S. galilaeus=Sarotherodon galilaeus, T. zilli=Tilapia zilli, O. aureus=Oreochromis aureus, T. simonis intermedia=Tristramella simonis intermedia, O. niloticus=Oreochromis niloticus,*

*O. mossambicus=Oreochromis mossambicus, P. schwanenfeldii=Puntius schwanenfeldii*

### Prevalence of TiLV in tissue

**Figure-2:**
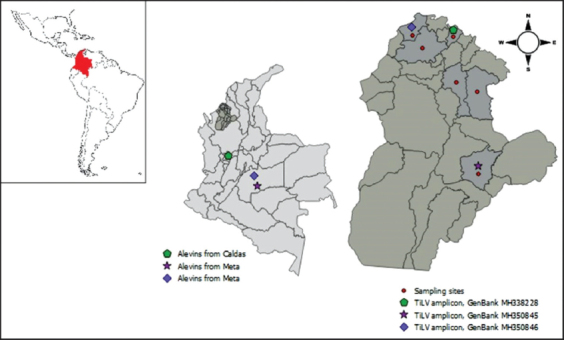
Sampling sites, origin of fingerlings, and the distribution of tilapia lake virus amplicons detected in the department of Córdoba [Source: The map was made using QGIS 2.18.22 ¨Las Palmas¨ Program, Spain].

TiLV was detected in 18 of 28 (64.3%) brain samples collected from Momil (n=8), Buenavista (n=5), and San Bernardo del Viento (n=5); in 13 of 21 (61.9%) spleen samples collected from Momil (n=6), Buenavista (n=5), and San Bernardo del Viento (n=2); and in 10 of 28 (35.7%) liver samples collected from Momil (n=2), Buenavista (n=3), and San Bernardo del Viento (n=5) (Figure-1). Fish collected from Momil came from the department of Caldas in the Andean mountains of Colombia, and the fingerlings collected from Buenavista and San Bernardo del Viento came from the department of Meta, an area near the Venezuelan border (Figure-2).

### Clinical presentation

**Figure-3:**
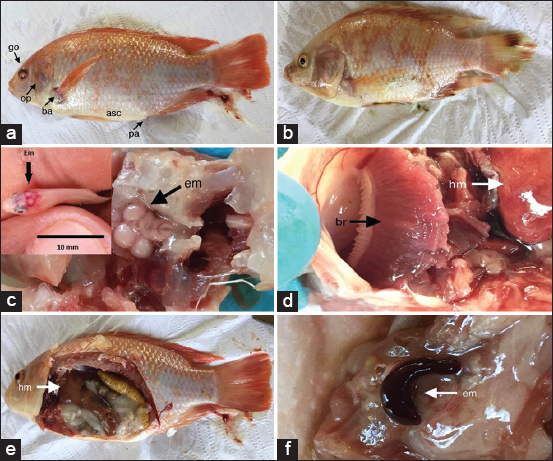
(a) Red hybrid tilapia with hemorrhagic eyeball (go), hemorrhagic operculum (op), the base of hemorrhagic fins (ba), ascites (asc), and congestive anal pore (pa). (b) Red tilapia (Oreochromis sp.) with discoloration and diffuse hemorrhagic congestion on the skin, nostril, and base of fins, fin necrosis, (c) red hybrid tilapia with encephalomegaly (em) in adult and fingerling (Em). (d) Fused and necrotic gills (br); hepatomegaly (hm), (e) red hybrid tilapia with hepatomegaly (hm) (female ovate). (f) Splenomegaly (em). (Photos from one of the authors A. Vallejo-Isaza).

The fish collected from the three farms with TiLV exhibited clinical signs such as lethargy, anorexia, thin fish with empty stomachs and intestines, visceral congestion (ascites), hepatomegaly, inflammation and rupture of the gallbladder, pale body, eye lesions, eroded fins, and splenomegaly (Figure-3).

### Environmental variables

During disease outbreaks in the fish farms of Momil-1, Buenavista, and San Bernardo del Viento, the average temperatures were 27.5°C, 32.0°C, and 28.0°C, respectively. The average dissolved oxygen contents were 5.0 mg/L in Momil-1, 5.85 mg/L in Buenavista, and 9.0 mg/L in San Bernardo del Viento. In the Momil-1 fish farm, the concentration of nitrite during the outbreak period was 0.75 mg/L. Variables such as pH, ammonium, nitrites, water hardness, alkalinity, and chlorides showed no considerable variations on the farms.

### Nucleotide and amino acid analysis of TiLV segment 3 sequences compared with GenBank sequences

The six sequences obtained in this study demonstrated 97% consensus identity with the sequence of segment 3 of the TiLV strain detected in tilapia from Israel in 2014 (GenBank KU751816.1) [13]. Three of the six sequences were deposited in GenBank, with the access codes MH338228 (Momil), MH350845 (Buenavista), and MH350846 (San Bernardo del Viento). The sequences obtained in this study showed eight different nucleotide changes at positions 330, 412, 414, 489, 567, 573, 612, and 726, concerning the phylogenetically closest sequence of TiLV segment 3 (GenBank KU751816.1_Israel) and with other GenBank sequences (KJ605629_Israel, KY381578_Thailand, MF582636_India, MF502419_India, and KX631923_Thailand). The sequences of the strains isolated from Momil-1 and San Bernardo del Viento displayed different nucleotide changes at positions 327, 609, 747, and 659. The nucleotide change at position 412 of all sequences demonstrated a non-synonymous substitution that produced a change in amino acid 138 from glutamic acid to lysine (E138K). Sequencing of the San Bernardo del Viento strain revealed nine nucleotide changes at position 659; a non-synonymous substitution produced a shift in amino acid 220 from valine to alanine (V220A).

### Nucleotide distances

The sequence closest to Colombian strains based on nucleotide identity was segment 3 of the Israeli strain (GenBank KU751816.1). The lowest degree of divergence with the Israeli strain was shown by the Buenavista sequence (GenBank MH350845) (divergence=0.02462), and the maximum degree of divergence was shown by the sequence from Momil-1 (GenBank MH338228) (divergence=0.03155). The most significant divergence of this study’s sequences was observed with the Thai strain (GenBank KY381578). There was also a slight divergence between the sequences of the strains from Buenavista and San Bernardo del Viento (divergence=0.002193), and these two strain sequences, in turn, exhibited a higher degree of divergence with the Momil-1 strain. The most significant divergence between the sequences of the department of Córdoba was observed between Momil-1 and San Bernardo del Viento strains (divergence=0.008830). The two sequences of the same fish farm strains exhibited 100% identity (degree of divergence=0.0).

### Phylogenetic tree

**Figure-4:**
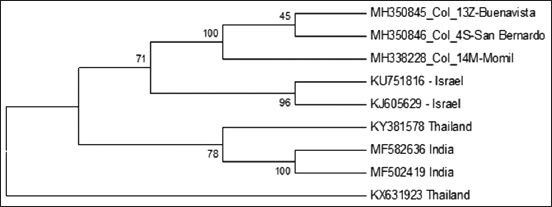
Comparing phylogenetic sequences of segment three of tilapia lake virus amplicons detected in Cordoba.

Phylogenetic reconstruction of TiLV was conducted using a 458 bp fragment of segment 3. For constructing the phylogenetic tree, the distances between homologous sequences were calculated using the Kimura 2-parameter model. The phylogenetic tree was constructed using the maximum likelihood method. The confidence values for the resulting tree´s branches were determined by bootstrap analysis with 1000 repetitions.. Three sequences were obtained in this study and six downloaded from GenBank were confronted. The phylogenetic analysis confirmed the relationship between the strains of the department of Córdoba and the strains of Israel (GenBank KU751816.1) (Figure-4).

## Discussion

This study demonstrated morbidity rates of 50-100% and mortality rates of 30-87% in each fish farm. The prevalence of TiLV infection is low and increases when massive mortalities are observed [14,15,30]. Results of the present study revealed 27.27% (18/66) prevalence of TiLV, indicating a more significant prevalence than that reported in Peru [21] at 14.8% (4/27) but lower than that reported in Ecuador [11], which was 63% (17/27).

It has been reported that fish viral infections can be spread horizontally within the crop, with an infection rate of 100% in just 8 days [13]. In the present study, it was observed that healthy fish were not infected with TiLV, even when cohabiting with infected fish in the majority of cases. This could be because fish that survived initial infection with TiLV developed immunity to the virus [13]. A recent study showed that tilapia that survived TiLV infection develop protective immunity that prevents subsequent infection [31]. The farmed fish in the present study exhibited a wide variety of clinical signs along with high mortality. The severity of the disease might be related to other pathogens; however, none of the farms considered infections with pathogens other than TiLV. Coinfection of TiLV and *Aeromonas veronii* in red tilapia was reported in 2018 by a previous study, wherein it was presumed that the interaction between these two pathogens was synergistic, thus increasing the disease severity [17]. Another study also reported the coinfection of TiLV and *A. veronii* in addition to *Aeromonas enteropelogenes* and *Aeromonas hydrophila* in 2017 [27]. Moreover, a recent study reported that coinfection of TiLV and *A. hydrophila* increased mortality and worsened disease severity in tilapia (*Oreochromis* spp.) [32]. In the present study, TiLV was detected in four healthy fish. Similar results have been reported in apparently healthy tilapia, wherein the authors considered this condition healthy at a low viral load but with a potential for transmission [21,33].

The mortality rates in the adult specimens of hybrid red tilapia (*Oreochromis* spp.) collected from the farms of Momil-1 and Buenavista were 85% and 87%, respectively, in this study, which is consistent with the previous reports [9,13]. In San Bernardo del Viento´s fish farm, a mortality rate of 20% was detected in red tilapia fingerlings, similar to that reported by Dong *et al*. [9].

In the present study, the liver, spleen, and brain tissue samples collected from fish with TiLV infection showed the disease´s clinical signs. Del-Pozo *et al*. [24] described the brain and liver as target organs of the virus; specifically, syncytial hepatitis has been described as a classic pathological finding of infection. Other authors have also described the brain as a target organ for TiLV [15,16,34]. We also observed that TiLV was present in 100% of the brain samples of tilapia with clinical signs of disease from the three positive farms, which allows confirming the tropism of the virus by this tissue. However, other authors did not detect TiLV in brain tissues [28]. Our results were inconsistent with those reported by Mugimba *et al*. [28], who did not detect TiLV in the brain tissue. However, Dong *et al*. [9] concluded that TiLV could be detected in the brain, gills, kidneys, liver, and spleen, and, more recently, it was also detected in reproductive organs [35]. On the other hand, we can argue that TiLV can cause a multisystemic infection, which may vary according to age, disease stage, and probably environmental aspects.

The genetic variation of TiLV observed in our study and other studies [9,25,36] could be related to the segmented genome’s feature. The genome allows mutations due to mixed infections caused by different viruses from different vertebrate animals, including human beings [37]. In the present study, it was observed that the variation of nucleotides at position 412 of the Colombian sequences showed a change at position 130 of the amino acid sequence of the protein. These changes in both nucleotides and amino acids of the TiLV strains detected within the same country were described for segments 1, 5, and 9 in the TiLV strains detected in Thailand [9,30].

TiLV infections exhibit differences in clinical presentation and organotropism, which could be associated with nucleotide mutations that can affect genome functions, proteins, and receptor specificity. This phenomenon would imply that there are genetic variants of TiLV with positive tropism for different tissues, as reported for the infectious salmon anemia virus with variations in virulence and cell tropism [38-40].

This study´s TiLV sequences revealed 95% identity with the TiLV segment 3 described in India (GenBank MF574205.1) [16]. However, it exhibited 97% identity with segment 3 of the Israeli strain (GenBank KU751816.1) [25]. Analysis of the TiLV genome´s partial sequences from different countries has revealed genetic variations [9,25,36]. Therefore, it is possible that the sequences obtained in this study also show nucleotide variations compared to those of the strains of Israel registered in GenBank. Our study results suggest that segment 3 of TiLV strain detected in Colombia is highly similar to that of TiLV segment 3 of Israel; however, the entry route remains unknown.

## Conclusion

To the best of our knowledge, this is the first report from the department of Córdoba confirming the molecular detection of TiLV. Moreover, this study demonstrates some possible foci of TiLV infection in Colombia. Analysis of segment 3 of the TiLV genome revealed new mutations, which could control the TiLV outbreaks in Colombia.

## Data Availability Statement

Consensus DNA sequences are available at the National Center of Biotechnology Information (Accession Numbers MH338228, MH350845, and MH350846).

## Authors’ Contributions

HC: Designed the study, writing of the manuscript, and analyzed the data. AV, LR, CG and AC: Helped in the practical experiment, drafted the manuscript and analyzed the data. SM: Helped in the design of the study, supervised, and drafted the manuscript. All authors read and approved the final manuscript.

## Competing Interests

The authors declare that they have no competing interests.
